# Long-chain organic molecules enable mixed dimensional perovskite photovoltaics: a brief view

**DOI:** 10.3389/fchem.2023.1341935

**Published:** 2024-01-11

**Authors:** Xianfang Zhou, Taomiao Wang, Xiao Liang, Fei Wang, Yan Xu, Haoran Lin, Ruiyuan Hu, Hanlin Hu

**Affiliations:** ^1^ Hoffmann Institute of Advanced Materials, Postdoctoral Innovation Practice Base, Shenzhen Polytechnic University, Shenzhen, China; ^2^ Jiangsu Provincial Engineering Research Center of Low Dimensional Physics and New Energy, School of Science, Key Laboratory for Organic Electronics and Information Displays, Institute of Advanced Materials (IAM), Jiangsu National Synergistic Innovation Center for Advanced Materials (SICAM), Nanjing University of Posts and Telecommunications, Nanjing, China

**Keywords:** long-chain organic molecules, 1D/3D mixed-dimensional perovskite, perovskite solar cell (PSC), long-term stability, low dimensional perovskite

## Abstract

The remarkable optoelectronic properties of organometal halide perovskite solar cells have captivated significant attention in the energy sector. Nevertheless, the instability of 3D perovskites, despite their extensive study and attainment of high-power conversion efficiency, remains a substantial obstacle in advancing PSCs for practical applications and eventual commercialization. To tackle this issue, researchers have devised mixed-dimensional perovskite structures combining 1D and 3D components. This innovative approach entails incorporating stable 1D perovskites into 3D perovskite matrices, yielding a significant improvement in long-term stability against various challenges, including moisture, continuous illumination, and thermal stress. Notably, the incorporation of 1D perovskite yields a multitude of advantages. Firstly, it efficiently passivates defects, thereby improving the overall device quality. Secondly, it retards ion migration, a pivotal factor in degradation, thus further bolstering stability. Lastly, the inclusion of 1D perovskite facilitates charge transport, ultimately resulting in an elevated device efficiency. In this succinct review, we thoroughly encapsulate the recent progress in PSCs utilizing 1D/3D mixed-dimensional architectures. These advancements encompass both stacked bilayer configurations of 1D/3D structures and mixed monolayer structures of 1D/3D. Additionally, we tackle critical challenges that must be surmounted and offer insights into the prospects for further advancements in this domain.

## 1 Introduction

The recent rapid development of research on organometal halide perovskite solar cells (PSCs) has made them become one of the most promising photoelectric materials to alleviate the energy crisis and global climate issues due to their economical cost, facilitated large-scale fabrication and, most of all, excellent photoelectric properties such as high absorption coefficient, long carrier diffusion length and broad-range absorption (Bai et al., 2019; Jeong et al., 2020; Bu et al., 2021; Jeong et al., 2021; Min et al., 2021; Yoo et al., 2021; Li et al., 2022a; Li et al., 2022b; Wang et al., 2022b; Ni et al., 2022). In the past decade, the power conversion efficiency (PCE) of PSCs has significantly improved to 26.1% (Best, 2023), which is comparable to commercial solar cells (Kojima et al., 2009; Min et al., 2021). However, despite their considerable potential in next-generation solar cells, further improvement of PSCs for practical application still faces several challenges, especially the unreliable stability in complex and harsh working environments due to the intrinsic structure of 3D perovskites that are extensively studied, making them sensitive when exposed to UV light, moisture, heat, and oxygen (Wang et al., 2016; Wang et al., 2017; Heo et al., 2019; Yang et al., 2020b; Nazir et al., 2022). To address this critical issue, the introduction of one-dimensional (1D) perovskite, constantly named as perovskitoid (Milić et al., 2019), has been regarded as an ideal alternative due to their unique characteristics such as structural diversity, multifunctional properties, and extraordinary environmental stability.

Generally, the chemical formula of 3D organometal halide perovskites is ABX_3_, in which A represents small cations such as methylammonium cations (MA^+^), Formamidine cations (FA^+^) and cesium cations (Cs^+^), B stands for bivalent metal cations (e.g., Pb^2+^, Sn^2+^) and X is halide anion (e.g., Cl^−^, Br^−^, I^−^), the typical structure of 3D perovskite can be described as the [BX_6_]^4-^ octahedra surrounded by A-site cations are corner-sharing with each adjacent one in all three dimensions to form octahedral framework, therefore 3D perovskites are classic bulk materials (Lin et al., 2018; Chu et al., 2021; Yao et al., 2021). It is worth noting that in order to maintain stable bulk structure, the radius of the A-site cations is strictly limited according to tolerance factor (Li et al., 2016; Bartel et al., 2019). By comparison, the utilization of large cations occupying A-site in 1D perovskites expands the lattice configuration, in the meantime the adjacent [BX_6_]^4-^ octahedra may not only be corner-sharing, but also be edge-sharing or face-sharing to form linear perovskite chains with “shoulder to shoulder” arrangement (Stoumpos et al., 2017; Zhou et al., 2017; Hong et al., 2018). The intrinsic high formation and low self-doping energies can remarkably suppress the occurrence of Schottky/Frenkel defects, thus inhibiting the ion migration. In addition, the presence of large hydrophobic cations efficiently prevents the lattice structure from moisture invasion, resulting in superior long-term stability of 1D perovskites (Fu et al., 2019; Kim and Seok, 2020; Paek et al., 2020). Nevertheless, the application of pure 1D perovskites in photovoltaic field suffers from a series of limitations, such as wide optical bandgap, high exciton binding energy caused by natural quantum wells, poor charge conductivity and low film quality (Koh et al., 2016; Yuan and Huang, 2016; Qian et al., 2017; Yang et al., 2018; Yang et al., 2022; Chen et al., 2023a; Zheng et al., 2023) Therefore, the 1D/3D mixed-dimensional perovskites have been extensively studied to combine the excellent photoelectric performance of 3D perovskites and the reliable long-term stability of 1D perovskites.

In this review, we summarize the recent progress on 1D/3D mixed-dimensional PSCs. We systematically classify them into 1D/3D stacked bilayers and 1D/3D mixed-dimensional layers according to the distribution of 1D perovskites. In particular, various functional effects or mechanisms owing to the incorporation of diverse 1D perovskite are stated. Finally, we carry on a brief summary and point out some perspectives toward further improving stability and efficiency of mixed-dimensional PSCs.

## 2 1D perovskite

**FIGURE 1:**
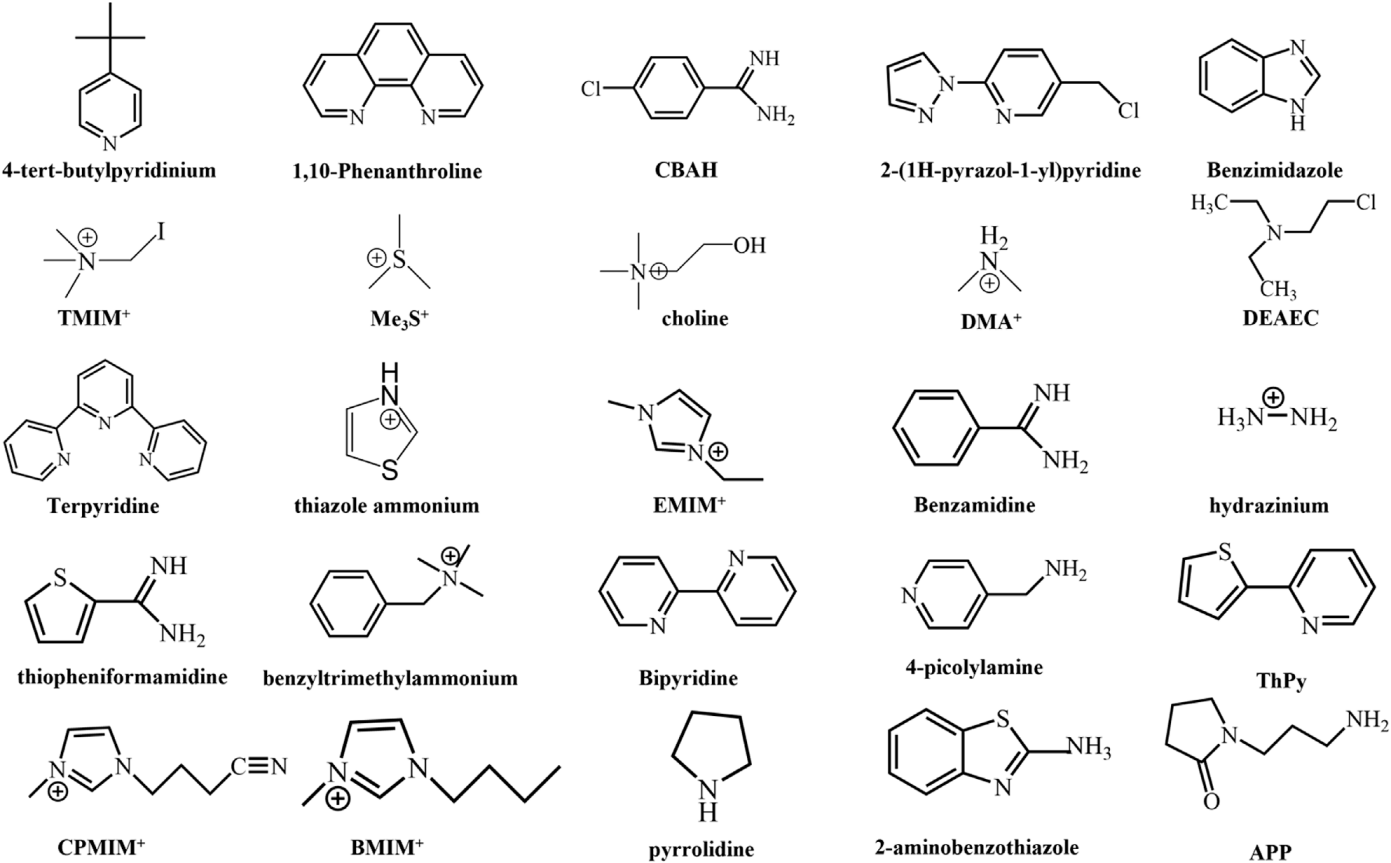
Molecular structures of A-site cations of organic small molecules used as 1D perovskite.

Typically, rigid and bulk organic cations are incorporated within the perovskite structure to serve as spacers, prompting the assembly of [BX_6_]^4−^ octahedra into a chain arrangement to form the 1D perovskite structure. The bulk organic cations that occupy the [BX_6_]^4−^ octahedral chain predominantly encompass aromatic amines, pyridinium, and so on (Chen et al., 2023b; Zhao et al., 2023). In this mini review, Figure 1 illustrates a range of A-site cations capable of inducing the formation of 1D perovskite structures. Attributed to the distinctive structure of 1D perovskite, where octahedral chains are isolated within rigid ammonium groups, carriers encounter confinement within the chain (Ge et al., 2022; Gao et al., 2023). This confinement results in elevated exciton binding energy and notable quantum confinement effects, which might not appear suitable for PSC applications. However, numerous reports have illustrated that when integrated into 1D/3D heterojunctions, these 1D perovskites can effectively function as passivation layers, thereby reducing interface defect density (Zhan et al., 2021a; Liu et al., 2023). Simultaneously, the inorganic [BX_6_]^4−^ octahedral chain is enveloped by rigid organic cations in 1D perovskites, resulting in a theoretically more environmentally stable structure than 3D perovskites (Zhu et al., 2022). This inherent stability is beneficial for enhancing the relative stability of PSC devices. The application of 1D/3D heterojunction structures in PSCs has been extensively explored and will be discussed in intricate detail below.

## 3 1D/3D stacked bilayer

**FIGURE 2:**
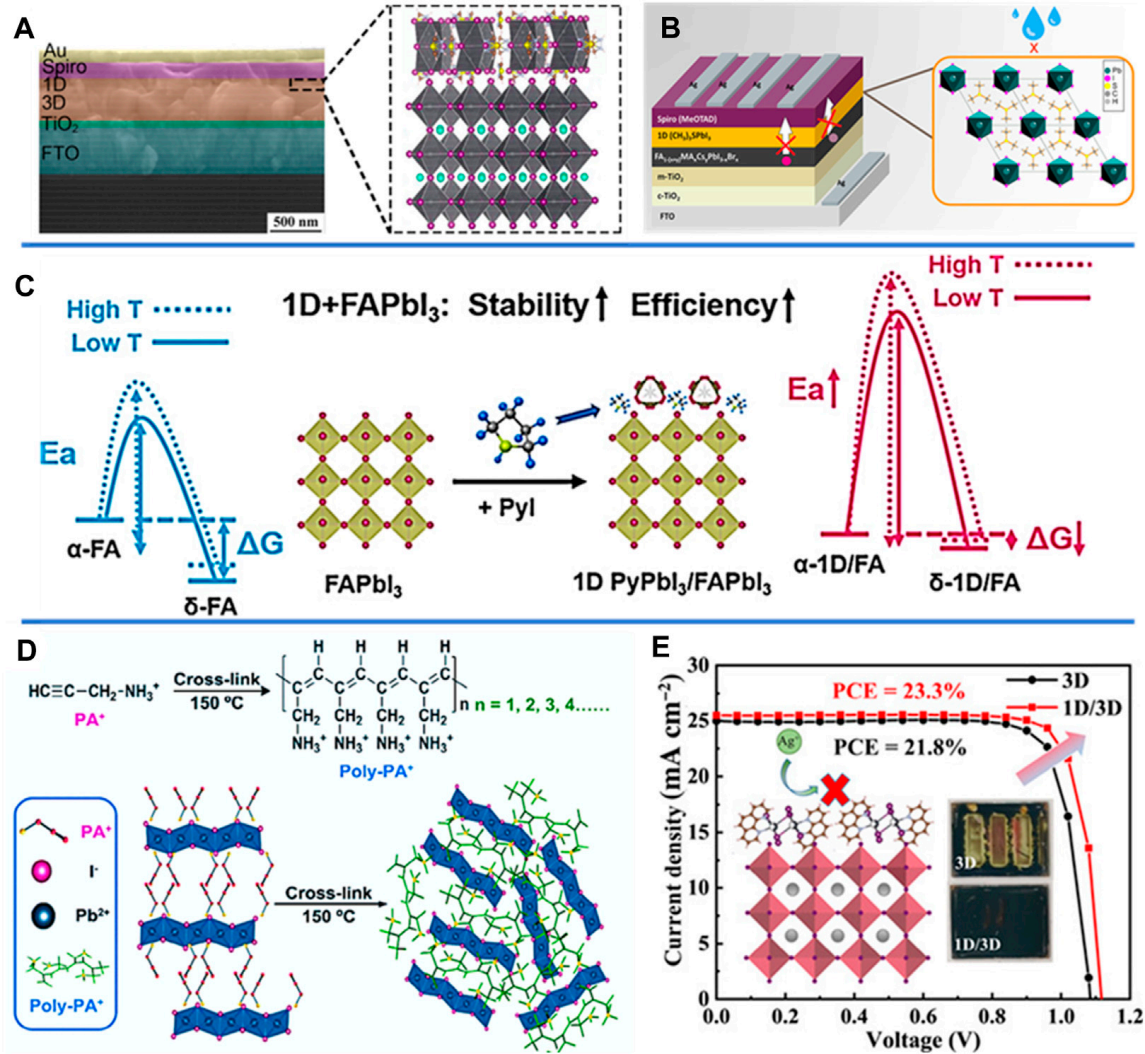
**(A)** Schematic illustration of crystal structure of TA_+_ based 1D/3D stacked perovskite. **(B)** Schematic diagram of device incorporated with 1D (CH_3_)_3_SPbI_3_. **(C)** The thermodynamic and kinetic analysis of 3D perovskite and 1D/3D perovskite phase transition process. **(D)** Schematic illustration of the cross-linking progress of PA^+^ in 1D perovskitoid. **(E)** *J-V* curves and apparent images of control and phen modified 1D/3D devices.

The bilayer multidimensional PSCs which contain pristine 3D perovskites layer and 1D perovskites passivation layer fabricated by posttreatment on top of 3D layer are considered to be an effective strategy. In 2019, Gao’s group (Gao et al., 2019) modified 3D perovskite by depositing thiazole ammonium (TA) through a gas-pump drying-assisted two-step process. More important, the introduction of a 1D capping layer effectively passivates 3D perovskite films, leading to enhanced charge transport, prolonged carrier lifetime, and prevention of iodide ion migration within the 3D perovskite. Moreover, the resultant device exhibited significant efficiency and superior environmental stability (Figure 2A). Elsenety et al. (2020) applied a 1D (CH_3_)_3_SPbI_3_ extra top layer *via* spin-coating onto the perovskite film (Figure 2B). This additional layer functions as a barrier, effectively hindering ionic migration and charge carrier recombination. Consequently, this barrier layer significantly enhances the stability of non-sealed devices, demonstrating improved performance under both ambient conditions and light-induced stress. Xu et al. (2020) address both the phase stability and energy efficiency concerns of FA-based PSCs by treating the perovskite surface with pyrrolidinium hydroiodide (PyI) salts. This treatment results in the formation of a 1D perovskite structure (PyPbI_3_) stacked onto the original 3D perovskite, revealing that the temperature-dependent phase transition activation barrier is heightened upon the creation of the 1D/3D structure *via in-situ* XRD measurements (Figure 2C). This innovative 1D/3D bilayer configuration holds the potential to be employed in PSCs to bolster both phase stability and photovoltaic performance. Yang et al. (2020a) introduce an innovative approach involving the *in situ* cross-linking of polymerizable propargylammonium (PA^+^) onto the surfaces and grain boundaries of 3D perovskite films to the formation of a 1D/3D perovskite heterostructure (Figure 2D). This passivation technique brings about notable enhancements in both interfacial carrier transport and the alleviation of residual tensile strain within the perovskite films. Consequently, the resulting devices achieve excellent photovoltaic performance. More recently, Chen et al. (2022a) effectively establishes a robust interface for perovskite films by employing 1D/3D mixed-dimensional engineering. Furthermore, the 1D capping layer acts as a diffusion barrier, curtailing ion migration and safeguarding the vulnerable 3D perovskite from moisture-induced degradation. The photovoltaic device achieves a high PCE and improved longterm stability (Figure 2E).

**FIGURE 3:**
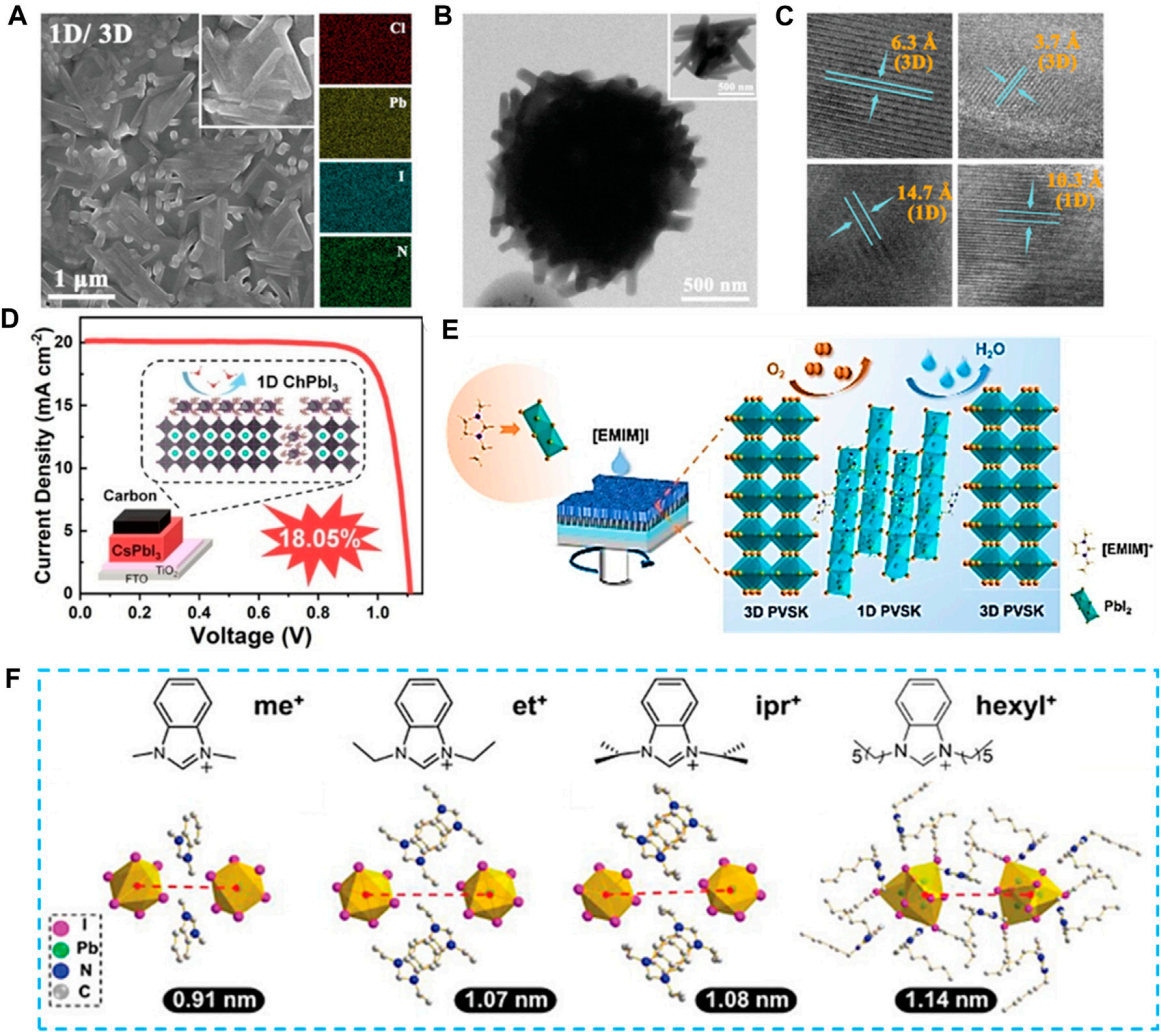
**(A)** Top-surface SEM and EDS images of the CBAH-treated perovskite films. **(B,C)** High-resolution TEM images of the CBAH-treated perovskite film. **(D)** ChPbI_3_ was formed via ChI treatment of CsPbI_3_ surface, creating a 1D/3D heterostructure that substantially enhances photovoltaic performance. **(E)** The ionic liquid [EMIM]I was introduced at the 3D perovskite surface to enhance device photovoltaic performance. **(F)** Crystal structures and corresponding front-view crystal structures of 1D perovskites.

Wang et al. (2022a) employed 4-chlorobenzamidine hydrochloride (CBAH) as a spacer to facilitate the development of an orientationally crystallized nanorod-like 1D perovskite layer atop the 3D perovskite surface. Further structural analyses reveal the coexistence of both 1D and 3D hybrid perovskite lattices within the nanorod-like passivation layer *via* SEM-EDS and TEM (Figures 3A–C). This coexistence effectively governs crystallization and morphology, consequently aiding in the enhancement of charge extraction and the suppression of charge recombination. Notably, the unencapsulated devices demonstrate elevated PCE, heightened thermal, moisture, and illumination stability. Zhang et al. (2023) confirmed ChI’s reaction with CsPbI_3_ to form a novel 1D ChPbI_3_ crystal phase, creating a 1D/3D heterostructure with CsPbI_3_, more effectively mitigating defects and substantially enhancing lifetime (Figure 3D). Remarkably, the 1D ChPbI_3_ suppressed δ-CsPbI_3_ deficiencies during formation, significantly bolstering CsPbI_3_ film stability. The final device achieved a certified record PCE of 18.05% for HTM-free inorganic C-PSCs. Meng et al. (2023) introduced the ionic liquid 1-ethyl-3-methylimidazolium iodide ([EMIM]I) to the 3D perovskite surface, reducing iodine vacancy defects and modulates band energy alignment, resulting in a substantial enhancement of the open circuit voltage (*V*_oc_) (Figure 3E). Consequently, the device achieves a high PCE and remarkable environmental and thermal stability. Chen et al. (2022b) introduced a novel type of benzimidazolium salt, N,N′-dialkylbenzimidazolium iodide to form 1D capping layer-topped 1D/3D structure (Figure 3F). This conformal interface modulation not only effectively stabilizes FACs-based perovskite films by impeding lateral and vertical iodide diffusion within devices or modules, thus ensuring excellent operational and environmental stability, but also establishes a proficient charge transport pathway through a well-designed 1D crystal structure.

## 4 1D/3D mixed monolayer

**FIGURE 4:**
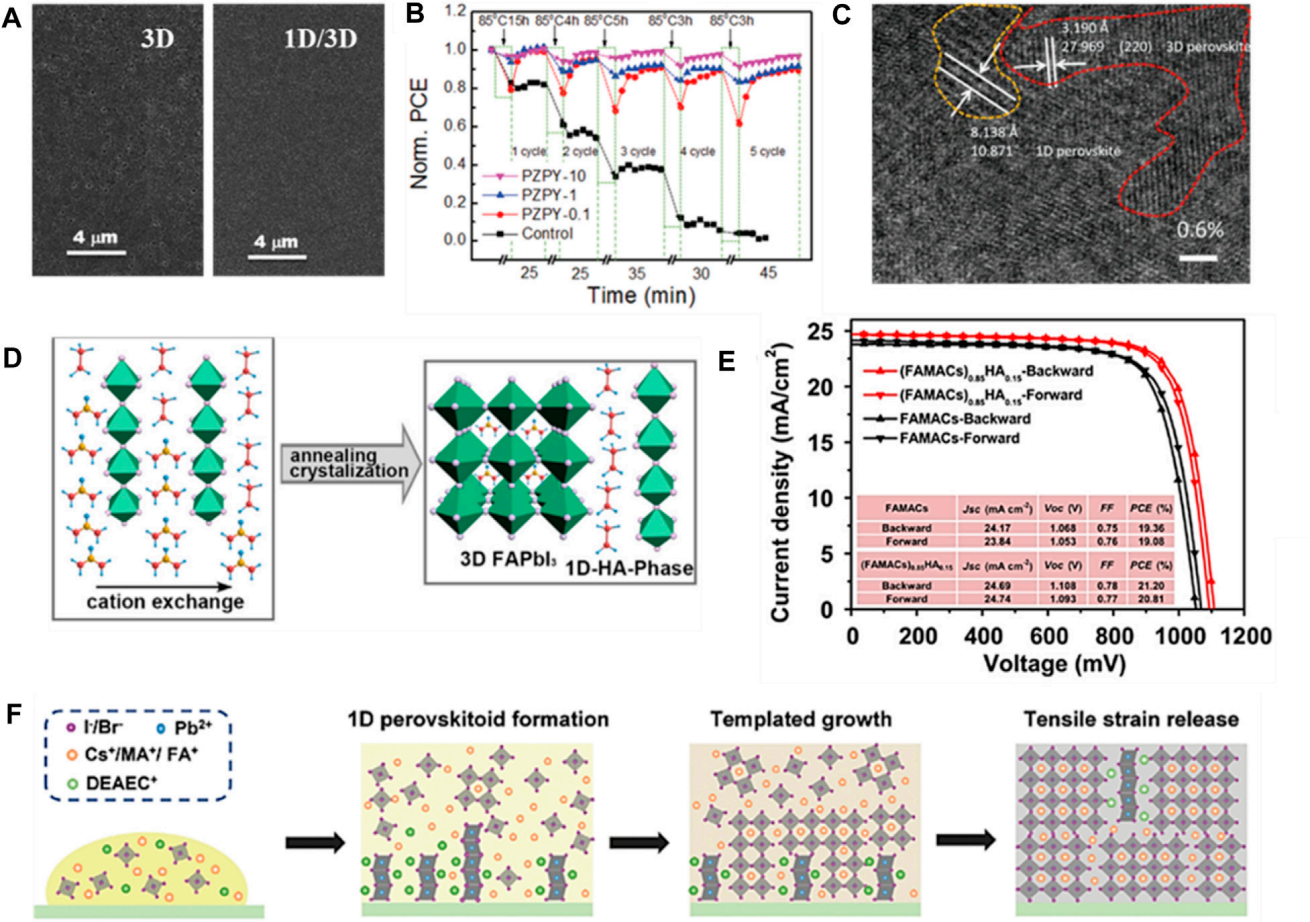
1D/3D mixed monolayer structure. **(A)** SEM images of pristine and mixed dimensional perovskite films. **(B)** The long-term stability of the unencapsulated control and 1D/3D hybrid PSCs during temperature cycling. **(C)** HRTEM image of 1D/3D heterostructure perovskite film. **(D)** Schematic illustration of cation exchange process between HA^+^ and FA^+^ and **(E)** *J-V* curves of control and HA modified devices. **(F)** Schematic diagram of the template-induced crystallization evolution process of the 1D/3D perovskite film introduced with DEAECCl.

Apart from the 1D/3D stacked bilayer structure mentioned above, the mixed-dimensional perovskite monolayer is another typical structure of 1D/3D perovskite heterojunction, in which the 1D perovskitoid can be regarded as a specific additive to reduce bulk defect density, block ion migration, and prevent the collapse of 3D perovskite induced by moisture. The most common method to introduce 1D perovskitoid is mixing target ligands in perovskite precursor solutions; hence, it is constantly capable of regulating morphology by effecting the crystallization process of perovskite. Early as in 2016, Bi et al. (2016) incorporated 1,1,1-trifluoro-ethyl ammonium iodide (FEAI) into MAPbI_3_ to simultaneously enhance the stability and efficiency. Moreover, the apparent defects which widely distributed on the surface of MAPbI_3_ has been obviously reduced after the introducing of FEAI *via* SEM (Figure 4A). The optimal device exhibited a PCE of 18% and long-term stability (Figure 4B). Fan et al. (2018) introduced 2-(1H-pyrazol-1-yl)pyridine (PZPY) into 3D perovskites, enabling the creation of 1D/3D hybrid perovskite materials. The 1D/3D hetero-structural exhibit the remarkable ability to significantly extend photoluminescence decay lifetime and suppress charge carrier recombination. Crucially, the fabricated 1D/3D PSCs showcase a thermodynamic self-healing capacity, attributed to the flexible 1D perovskite’s ability to obstruct the ion-migration pathways of A-site ions. Zhang et al. (2021) demonstrate the formation of 1D/3D mixed perovskites by integrating room temperature 1D ferroelectric TMIMPbI_3_ with 3D perovskite, displaying notable ferroelectric properties. HR-TEM analysis confirmed that the incorporation of the 1D perovskite into the precursor results in the formation of a 1D/3D mixed perovskite film. The 1D phase is uniformly dispersed throughout the film without noticeable phase separation, underscoring that the 1D/3D mixed perovskite represents an integral entity with a shared PbI_6_ octahedra backbone (Figure 4C). Yu et al. (2021) introduced hydrazinium cation (HA^+^: NH_2_NH_3_^+^) into perovskite layer. The robust hydrogen bonding of HA^+^ elevates perovskite crystallinity and grain size (Figures 4D, E). This engenders a 1D/3D hybrid dimension structure, bolstering the photovoltaic performance of PSCs. Kong et al. (2021) utilize 2-diethylaminoethylchloride cations (DEAEC^+^) as templates to induce a 1D@3D perovskite structure (Figure 4F), resulting in smoother surface texture, extended charge-carrier lifetime, reduced residual tensile strain, and minimized surface-defect density in the perovskite film. This approach yields highly efficient and stable 1D@3D perovskite solar cells (PSCs) with remarkable reproducibility. Zhan et al. (2021a) investigated a novel 1D/3D perovskite material through the introduction of a bulk benzimidazole cation (Bn^+^) organic salt. Bn^+^ induces controlled crystalline growth of 3D perovskite with preferred orientation while creating spatially distributed 1D BnPbI_3_ perovskite within the 3D perovskite film. The 1D/3D perovskite effectively curbs electro-strictive responses and imbalanced charge carrier extraction, cultivating an inherently stable lattice with fortified ionic bonds and reduced excess charge carriers. Consequently, ion migration tendencies within the p-i-n 1D/3D PSC are significantly mitigated under operational conditions. Wei et al. (2022) introduced a functional ionic liquid, 1-ethyl-3-methylimidazolium trifluoroacetate (EMIMTFA), where EMIM^+^ interact with PbI_2_ to establish a stable 1D perovskite, and anions TFA^−^ passivate the perovskite surface. EMIM^+^ cations are distributed not only on the top surface and within the bulk phase but also accumulate at the buried interface of the 3D perovskite film, resulting in a multi-level distribution. This distribution effectively passivates distinct defect states, notably reducing non-radiative recombination.

**FIGURE 5:**
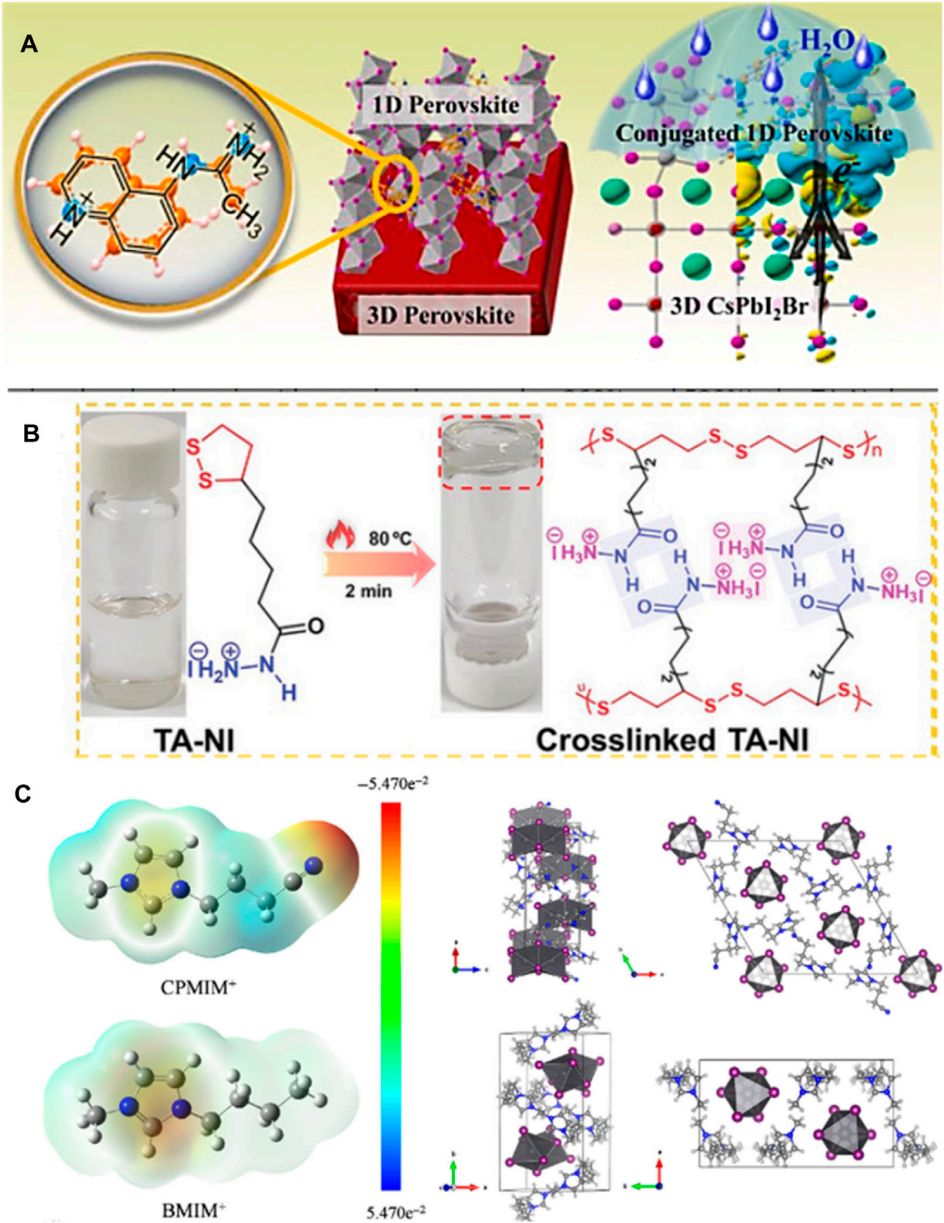
**(A)** A-site quinoline organic groups and 1D–3D heterojunction. **(B)** TA-NI before and after cross-linking: the state in solvent and chemical structures. **(C)** ESP distribution maps and structural arrangements of ions CPMIM^+^ and BMIM^+^.

Long et al. (2021) introduced aminoquinoline to create a new 1D perovskite with closely arranged distorted octahedra and orderly distributed quinoline (Aq) organic groups (Figure 5A). Incorporating this 1D AqPbI_3_(II) perovskite into perovskite improves carrier transport and hydrophobicity due to its unique structure and lattice-matching heterojunction. The resulting cell increased *V*_oc_, PCE, and stability. Chen et al. (2023c) developed a cross-linkable monomer [5-(1,2-dithiolan-3-yl)] pentanehydrazide hydroiodide (TA-NI) with specific building blocks, including dynamic covalent disulfide bonds, H-bonds, and ammonium functional groups (Figure 5B). This cross-linked TA-NI possesses elastomeric properties and can autonomously self-heal at room temperature. It also forms cross-links during perovskite film growth, attaching to grain boundaries and repairing mechanical-stress-induced cracks in the perovskite film. Furthermore, the ammonium functional group can interact with PbI_6_^4−^ to create a 1D perovskite, which reduces residual tensile strain in the perovskite, mitigates lattice mismatch, and inhibits ion migration. Wang et al. (2023) introduced an IL called 1-(3-cyanopropyl)-3-methylimidazolium chloride (CPMIMCl) to induce an in-plane preferred orientation of 1D perovskite in 3D perovskite thin films through a sequential deposition process. The crystal structure of 1D perovskite is shown in Figure 5C. This in-plane-oriented 1D CPMIMPbI_3_ resides at grain boundaries within 3D perovskite, resulting in high-quality films with larger grain sizes and enhanced defect passivation. Consequently, the devices exhibit enhanced photovoltaic performance. We summarize all PSCs based on 1D/3D mixed-dimensional structure in Table 1.

**TABLE 1:** Summary of the properties of PSCs based on 1D/3D mixed-dimensional structure.

3D perovskite	1D perovksite	Type	Device structure	PCE (%)	Stability	Year	Ref.
(MA, FA) PbI_3_	TAPbI_3_	Stacked	FTO/TiO_2_/Perovskite/Spiro-OMeTAD/Au	18.97	∼300 h (∼70% RH)	2019	Gao et al. (2019)	
FA_0.9_Cs_0.07_MA_0.03_Pb (I_0.92_Br_0.08_)_3_	EAPbI_3_	Stacked	FTO/TiO_2_/Perovskite/Spiro-OMeTAD/Au	22.3	>550 h (N_2_)	2019	Elsenety et al. (2020)	
(FA/MA/Cs) PbI_3−x_Br_x_	(CH_3_)_3_SPbI_3_	Stacked	FTO/c-TiO_2_/m-TiO_2_/Peroskite/Spiro-OMeTAD/Ag	16.67	∼350 h (∼30% RH)	2020	Xu et al. (2020)	
MAPbI_3_	PyPbI_3_	Stacked	FTO/c-TiO_2_/m-TiO_2_/Peroskite/Spiro-OMeTAD/Au	16.65	100 days (30%–65% RH)	2019	Yang et al. (2020a)	
FA_0.9_Cs_0.07_MA_0.03_Pb (I_0.92_Br_0.08_)_3_	PyPbI_3_	Stacked	ITO/SnO_2_/Perovskite/Spiro-OMeTAD/Ag	19.62	∼18 days (∼65% RH)	2020	Chen et al. (2022a)	
FAPbI_3_	CBAH	Stacked	ITO/SnO_2_/3D FAPbI_3_-1D layer/Spiro-OMeTAD/MoO_3_/Ag	21.95	∼320 h (1.5G irradiation)	2021	Wang et al. (2022a)	
CsPbI_3_	ChPbI_3_	Stacked	Hole transport material (HTM)-free carbon-based CsPbI3 PSCs	18.05	∼120 h (1 sun irradiation)	2023	Zhang et al. (2023)	
FA_0.5_MA_0.5_PbI_3_	[EMIM]PbI_3_	Stacked	FTO/SnO_2_/FA_0.5_MA_0.5_PbI_3_/[EMIM]I/Spiro-OMeTAD/Au	19	∼1,320 h (∼30% RH)	2023	Meng et al. (2023)	
FA_0.9_Cs_0.1_PbI_3_	hexylPbI_3_	Stacked	ITO/SnO_2_/FA_0.9_Cs_0.1_PbI_3_/Spiro-OMeTAD/Au	24.3	∼500 h (∼60% RH)	2022	Chen et al. (2022b)	
MAPbI_3_	FEAPbI_3_	Mixed	FTO/c-TiO_2_/m-TiO_2_/Peroskite/Spiro-OMeTAD/Au	18	N/A	2016	Bi et al. (2016)	
Cs_0.04_MA_0.16_FA_0.8_PbI_0.85_Br_0.15_	PbBr_2_-PZPY	Mixed	FTO/TiO_2_/Perovskite/PTAA/Au	18.1	35 h (∼40% RH)	2018	Fan et al. (2018)	
(FAPbI_3_)_0.85_ (MAPbBr_3_)_0.15_	TMIMPbI_3_	Mixed	ITO/SnO_2_/Perovskite/Spiro-OMeTAD/Au	22.7	130 h (∼50% RH)	2021	Zhang et al. (2021)	
Cs_0.05_ (MA_0.17_FA_0.83_)_0.95_Pb(I_0.83_Br_0.17_)_3_	HAPbI_3_	Mixed	FTO/TiO_2_/Perovskite/Spiro-OMeTAD/Au	21.2	2,500 h (∼30% RH)	2021	Yu et al. (2021)	
(Cs_0.04_FA_0.86_MA_0.1_) Pb(I_0.9_Br_0.1_)_3_	DEAECPbI_3_	Mixed	ITO/SnO_2_/Perovskite/Spiro-OMeTAD/Ag	22.9	2,000 h (5%–10% RH)	2021	Kong et al. (2021)	
Cs_0.05_FA_0.81_MA_0.14_PbI_2.55_Br_0.45_	BnPbI_3_	Mixed	ITO/PTAA/Perovskite/C_60_/BCP/Ag	21.17	3,000 h	2021	Zhan et al. (2021a)	
MAPbI_3_	EMIMPbI_3_	Mixed	ITO/c-TiO_2_/SnO_2_/Perovskite/Spiro-OMeTAD/Ag	22.14	120 h (∼35% RH)	2021	Wei et al. (2022)	
CsPbI_2_Br	Aq-PbI_3_	Mixed	FTO/TiO_2_/Perovskite/Spiro-OMeTAD/Ag	16.1	∼1,300 h (∼30% RH)	2021	Long et al. (2021)	
FA_0.92_MA_0.08_PbI_3_	TAPbI_3_	Mixed	PET/ITO/SnO_2_/Perovskite/Spiro-OMeTAD/Au	23.84	3,000 h (∼30% RH)	2023	Chen et al. (2023c)	
(FA/MA/Cs) PbI_3_	CPMIMPbI_3_	Mixed	ITO/SnO_2_/perovskite/Spiro-OMeTAD/Au	24.13	∼1,000 h (∼50% RH)	2023	Wang et al. (2023)	

## 5 Conclusion and outlooks

The excellent photoelectric properties of 3D perovskite and reliable stability of 1D perovskite have attracted tremendous attention on researches of 1D/3D mixed-dimensional perovskite. In the past few years, numerous precious outbreaks have been achieved already, in this review we divide they into two typical structures according to the distribution of 1D perovskite and briefly state the recent research progress of each one. Overall, the beneficial improvements of the introduction of 1D perovskite can be summarized as reduced defects, inhibited ion migration, promoted charge transport and, most of all, enhanced long-term stability. As mentioned previously in this review, the impressive high PCE of devices based on 1D/3D mixed-dimensional perovskite can be realized by applying reasonable and appropriate strategies, and most of them only get small losses of initial efficiency after aging under complex conditions for long period. In addition, the incorporation of 1D perovskite may leads to various extra functional effects or special configurations due to the diversity of additional ligands, such as the cross-linked structure, thermodynamic self-healing ability, ferroelectricity and so on.

Even though great improvements have been achieved in this field, substantial issues that severely restrict the further developments and potential applications of 1D/3D mixed-dimensional PSCs need to be addressed urgently. The major problem is the unsatisfied device efficiency compared to that of the state-of-the-art PSCs (26.1%). This is mainly attributed to the intrinsic poor and anisotropic charge conductivity of 1D perovskite, hence the further exploration for new ligand that possess the capability of forming 1D perovskite and better natural characteristics is essential. Secondly, most researches are focus on small-area films and devices, which are definitely far away from available practical applications. Even if appreciable PCE of small-area devices have been realized, it is not convincing for preparation of large-area devices considering the difficulty of fabricating large area films. Therefore, the exploration on preparation methods toward high quality large-area films is essential to meet the requirements in practical applications. Finally, the low-cost advantage of PSCs is always weakened by the high-cost of electron or hole transmission layer materials and electrode materials, developing budget alternative materials and feasible strategy to reduce the total cost is necessary. Apart from these three problems stated above, there are numerous challenges that the application of PSCs needs to face, nonetheless, we still consider 1D/3D mixed-dimensional strategy as prospective method to improve the efficiency and stability of PSCs simultaneously, thus leading to the large-scale application of PSCs in the future.
